# Supplementary material for: Exploring the Genotoxic Stress Response in Primed Orphan Legume Seeds Challenged with Heat Stress
Source: Genes (Basel). 2025 Feb 19;16(2):235. doi: 10.3390/genes16020235 (PMC11855731; doi:10.3390/genes16020235)
Supplement: Supplementary file 1 [file genes-16-00235-s001.zip › genes-3483614-supplementary.pdf]

Supplemental information

Exploring the genotoxic stress response in primed orphan legumes seeds challenged with heat stress

Andrea Pagano, Conrado Jr. Dueñas, Nicolò Bedotto, Amine Elleuch, Bassem Khemakhem, Hanen El Abed, Eleni Tani, Maro Goufa, Dimosthenis Chachalis, Alma Balestrazzi

Supplementary Table S1. List of oligonucleotide primers used for *q*RT-PCR analyses. For each oligonucleotide set, PCR efficiency (E) is reported. *Ls*, *Lathyrus sativus*. *EF2, ELONGATION FACTOR 2. GAPDH, GLYCERALDEHYDE-3-PHOSPHATE DEHYDROGENASE. SOD, SUPEROXIDE DISMUTASE. APX, ASCORBATE PEROXIDASE. MT, METALLOTHIONEIN. OGG1, 8-OXOGUANINGLYCOSYLASE/LYASE. FPG, FORMAMIDOPYRIMIDINE-DNA GLYCOSYLASE. Lig, DNA LIGASE. TOP, DNA TOPOISOMERASE. TFIIS, TRANSCRIPTION ELONGATION FACTOR IIS. SPMS, SPERMINE/SPERMIDINE SYNTHASE. SPDS, SPERMIDINE SYNTHASE. 5.8S, 5.8S rRNA. 5.8S-IS, 5.8S rRNA-INTERSPACE. PCNA, PROLIFERATING CELL NUCLEAR ANTIGEN. SOG1, SUPPRESSOR OF THE GAMMA RESPONSE 1.*

**Gene** (Accession n°)	**Oligonucleotide Forward**	**Oligonucleotide Reverse**	**E**
LsEF2a (GBSS01000402)	5'-CCTCCAGTGGTCTTGCTTCC-3'	5'-TGATTTCATGGGTGGGGCTG-3'	1.8
LsGAPDH (GBSS01000223)	5'-GCTCCCAGTAAAGATGCTCCC-3'	5'-AGCCCTTCAACAATGCCAAAC-3'	1.8
LsSODa (GBSS01003115)	5'-TCTCCACTGGTCCTCACTTC-3'	5'-CCCTCCCGAGTATGGAATGA-3'	1.8
LsSODb (GBSS01001045)	5'-GTGGACAACAACAGCCCTTC-3'	5'-TGCCCCTGAGGATGAGACTA-3'	1.8
LsAPXa (GBSS01001446)	5'-TTCAGAGCTTGGCTTTGTGC-3'	5'-ACATCCAACAGAGCTGAGCA-3'	1.8
LsAPXb (GBSS01019041)	5'-GTAGCGGTAGAGGTCACTGG-3'	5'-GCGATGCACCTTGTTTAGCA-3'	1.8
LsMT1 (GBSS01000013)	5'-ATCAAAGTGGATCTTCGCCG-3'	5'-GTTGTGGAAGCAGTTGC-3'	1.8
LsMT2 (GBSS01000484)	5'-TCTTGCTGTGGTGGAAACTG-3'	5'-CTCAGATCCGACTCCCATGA-3'	1.8
LsOGG1 (GBSS01016977)	5'-TTGAGGGCTACCAAACAGC-3'	5'-CGTAGTGTGTTACGCCTGAG-3'	1.8
LsFPG (GBSS01018706)	5'-GCGAGCTGCAACAATGGTTT-3'	5'-TAACCAAGTGCATCGTCGCA-3'	1.8
LsLig1 (GBSS01022069)	5'-AGCAACCAAAGGTGTAGCAG-3'	5'-TGCTGCAACAACATCAGGAA-3'	1.8
LsTOP1 (GBSS01013769)	5'-TCGTAGTGTTTGGCTGAGGTG-3'	5'-CACGGAAGGCACGCTGATA-3'	1.8
LsTFIIS (GBSS01005128)	5'-GCTCAACAGTACCGACCAGA-3'	5'-GCAGTGACAGTGGAGTCTC-3'	1.8
LsSPSD (GBSS01000983)	5'-TCAGAGAACCAGCCAGGAAT-3'	5'-GGCACCAGAAAACACACTTCA-3'	1.8
LsSPMS (GBSS01003422)	5'-GTGTGAATGTCGGTGAGAGG-3'	5'-ATGCACTGAGCCACCATTTT-3'	1.8
Ls5.8SrRNA (AY839389)	5'- TCCCGTGAACCATCGAGTCT -3'	5'- CCCTCAACCTAATGGCATCG -3'	1.8
Ls5.8SrRNA-IS (AY839389)	5'- GTTTCCGTGCGGGCTGTG -3'	5'- CCCTCAACCTAATGGCATCG -3'	1.8
LsPCNA (GBSS01017540)	5'-GAATCCCGAGGTGTTCACT-3'	5'-TATGGCTAAGATGTTGAAGTGCG-3'	1.8
LsSOG1	5'-CTTTTCCTCCTCCCGCACT-3'	5'-GATTTTGGTGATGTCCGCTGG-3'	1.8

(GBSS01010054)			

Supplementary Table S2. Relative expression values of target genes in the tested experimental conditions in *L. sativus* Maleme-107 accession. For each gene, expression levels without common letters are significantly different (*p*-value < 0.05) as analyzed by one-way ANOVA and Duncan test. Values are expressed as mean ± standard error. DS, dry seeds; DB, seeds subjected to hydropriming and dry-back; UP, unprimed seeds; HP, hydroprimed seeds; NT, seeds subjected to 8 h imbibition at 25 °C without heat wave; HW, seeds subjected to 4 h imbibition at 25 °C followed by 4 h heat wave at 40 °C. *Ls*, *Lathyrus sativus*. *EF2*, *ELONGATION FACTOR 2*. *GAPDH*, *GLYCERALDEHYDE-3-PHOSPHATE DEHYDROGENASE*. *SOD*, *SUPEROXIDE DISMUTASE*. *APX*, *ASCORBATE PEROXIDASE*. *MT*, *METALLOTHIONEIN*. *OGG1*, *8-OXOGUANINGLYCOSYLASE/LYASE*. *FPG*, *FORMAMIDOPYRIMIDINE-DNA GLYCOSYLASE*. *Lig*, *DNA LIGASE*. *TOP*, *DNA TOPOISOMERASE*. *TFIIS*, *TRANSCRIPTION ELONGATION FACTOR IIS*. *SPMS*, *SPERMINE/SPERMIDINE SYNTHASE*. *SPDS*, *SPERMIDINE SYNTHASE*. *5.8S*, *5.8S rRNA*. *5.8S-IS*, *5.8S rRNA-INTERSPACE*. *PCNA*, *PROLIFERATING CELL NUCLEAR ANTIGEN*. *SOG1*, *SUPPRESSOR OF THE GAMMA RESPONSE 1*.

Gene	**DS**	**DB**	**UP-NT**	**UP-HW**	**HP-NT**	**HP-HW**
LsSODa	0.138 ± 0.00299ab	0.107 ± 0.00825c	0.153 ± 0.00125a	0.132 ± 0.00733b	0.0468 ± 0.00195d	0.0555 ± 0.00277d
LsSODb	0.958 ± 0.0448a	0.705 ± 0.0316b	0.598 ± 0.0177c	0.536 ± 0.0181c	0.272 ± 0.00958d	0.565 ± 0.0092c
LsAPX3a	0.0214 ± 0.0051b	0.0831 ± 0.00306a	0.0215 ± 0.00153b	0.0222 ± 0.00109b	0.0267 ± 0.001b	0.0236 ± 0.00165b
LsAPX3b	0.0138 ± 0.00228b	0.00827 ± 0.00136c	0.00708 ± 0.00107c	0.039 ± 0.0025a	0.00424 ± 0.000155c	0.00533 ± 6.33e-05c
LsMT1	0.13 ± 0.0144a	0.116 ± 0.018ab	0.076 ± 0.00879c	0.037 ± 0.00303d	0.122 ± 0.00751a	0.0829 ± 0.00569bc
LsMT2	2.13 ± 0.16b	2.38 ± 0.044a	1.27 ± 0.0587d	1.72 ± 0.0284c	0.503 ± 0.00634e	1.24 ± 0.0157d
LsSPDS	0.00448 ± 0.000344b	0.0104 ± 0.000632a	0.00977 ± 0.000279a	0.0105 ± 0.000676a	0.00495 ± 0.000524b	0.0044 ± 0.000248b
LsSPMS	0.00943 ± 0.00172d	0.014 ± 0.00117bc	0.0171 ± 0.00186ab	0.0197 ± 0.000875a	0.00838 ± 0.000411d	0.0113 ± 0.000347cd
LsSOG1	0.00069 ± 0.00015b	0.0014 ± 0.000354a	0.000101 ± 2.98e-05c	0.000132 ± 2.8e-05c	0.000803 ± 2.84e-05b	7.17e-05 ± 0c
LsOGG1	0.021 ± 0.00168c	0.00639 ± 0.00203e	0.0617 ± 0.00215a	0.0537 ± 0.00296b	0.0159 ± 0.000759cd	0.0136 ± 0.000313d
LsFPG	0.0107 ± 0.00204c	0.0138 ± 0.00154c	0.0567 ± 0.00295b	0.0956 ± 0.00767a	0.0146 ± 0.000705c	0.0122 ± 0.000586c
LsLig	0.00364 ± 0.000503e	0.0101 ± 0.0014e	0.0597 ± 0.000786b	0.0819 ± 0.00842a	0.0447 ± 0.00188c	0.0223 ± 0.000768d
LsTop1	0.0508 ± 0.00429b	0.0346 ± 0.0019c	0.0563 ± 0.0027b	0.0899 ± 0.00124a	0.0321 ± 0.00201c	0.0214 ± 0.00171d
LsTFIIS	0.0897 ± 0.00799b	0.0508 ± 0.00107b	0.168 ± 0.027a	0.204 ± 0.0149a	0.0547 ± 0.00967b	0.0825 ± 0.0187b
Ls5.8S	1240 ± 7.88c	2160 ± 115a	1740 ± 165b	601 ± 43.7d	245 ± 47.8e	529 ± 110de
Ls5.8S-IS	219 ± 10.1b	156 ± 6.45c	290 ± 14.5a	182 ± 8.43c	123 ± 6d	161 ± 2.44c
LsPCNA	0.0292 ± 0.0014d	0.0229 ± 0.000494d	0.11 ± 0.0089b	0.144 ± 0.00408a	0.0567 ± 0.000755c	0.0493 ± 0.00147c

Supplementary Table S3. Relative expression values of target genes in the tested experimental conditions in *L. sativus* Sofades accession. For each gene, expression levels without common letters are significantly different (*p*-value < 0.05) as analyzed by one-way ANOVA and Duncan test. Values are expressed as mean ± standard error. DS, dry seeds; DB, seeds subjected to hydropriming and dry-back; UP, unprimed seeds; HP, hydroprimed seeds; NT, seeds subjected to 8 h imbibition at 25 °C without heat wave; HW, seeds subjected to 4 h imbibition at 25 °C followed by 4 h heat wave at 40 °C. *Ls*, *Lathyrus sativus*. *EF2*, *ELONGATION FACTOR 2*. *GAPDH*, *GLYCERALDEHYDE-3-PHOSPHATE DEHYDROGENASE*. *SOD*, *SUPEROXIDE DISMUTASE*. *APX*, *ASCORBATE PEROXIDASE*. *MT*, *METALLOTHIONEIN*. *OGG1*, *8-OXOGUANINGLYCOSYLASE/LYASE*. *FPG*, *FORMAMIDOPYRIMIDINE-DNA GLYCOSYLASE*. *Lig*, *DNA LIGASE*. *TOP*, *DNA TOPOISOMERASE*. *TFIIS*, *TRANSCRIPTION ELONGATION FACTOR IIS*. *SPMS*, *SPERMINE/SPERMIDINE SYNTHASE*. *SPDS*, *SPERMIDINE SYNTHASE*. *5.8S*, *5.8S rRNA*. *5.8S-IS*, *5.8S rRNA-INTERSPACE*. *PCNA*, *PROLIFERATING CELL NUCLEAR ANTIGEN*. *SOG1*, *SUPPRESSOR OF THE GAMMA RESPONSE 1*.

Gene	**DS**	**DB**	**UP-NT**	**UP-HW**	**HP-NT**	**HP-HW**
LsSODa	0.108 ± 0.0062a	0.0438 ± 0.00774b	0.0886 ± 0.00642a	0.111 ± 0.00764a	0.0435 ± 0.000543b	0.0561 ± 0.0199b
LsSODb	1.79 ± 0.0604ab	0.934 ± 0.0639d	1.43 ± 0.0424bc	1.9 ± 0.061a	0.879 ± 0.0408d	1.24 ± 0.271cd
LsAPX3a	0.0244 ± 0.00165c	0.132 ± 0.00736a	0.0251 ± 0.000647c	0.047 ± 0.00144bc	0.0563 ± 0.00502bc	0.073 ± 0.0327b
LsAPX3b	0.0118 ± 0.00169b	0.0228 ± 0.00368a	0.0142 ± 0.00247b	0.0294 ± 0.00204a	0.00944 ± 0.0016b	0.00912 ± 0.000805b
LsMT1	0.0235 ± 0.00615b	0.21 ± 0.00903a	0.0127 ± 0.00491bc	0.000243 ± 8.98e-05c	0.00472 ± 0.00125c	0.0248 ± 0.00628b
LsMT2	1.89 ± 0.0349a	0.89 ± 0.0896d	1.48 ± 0.0553bc	1.58 ± 0.0646b	0.536 ± 0.0181e	1.31 ± 0.0644c
LsSPDS	0.00456 ± 0.000108	0.00418 ± 0.000745	0.00396 ± 0.00151	0.00324 ± 0.00117	0.00383 ± 0.000336	0.00559 ± 0.00154
LsSPMS	0.0105 ± 0.00117	0.0112 ± 0.00409	0.00786 ± 0.00238	0.0147 ± 0.00438	0.00479 ± 0.00106	0.00846 ± 0.00165
LsSOG1	0.00855 ± 0	0.065 ± 0.0278	0.0524 ± 0.00479	0.0812 ± 0.0119	0.0887 ± 0.0328	0.092 ± 0.0403
LsOGG1	0.0125 ± 0.0026cd	0.0109 ± 0cd	0.0387 ± 0.00244a	0.0328 ± 0.00493ab	0.00729 ± 0.00181d	0.0227 ± 0.00692bc
LsFPG	0.02 ± 0.00284b	0.0275 ± 0.00649b	0.0543 ± 0.00273a	0.0711 ± 0.0105a	0.0313 ± 0.0025b	0.0688 ± 0.012a
LsLig	0.00932 ± 0.00165c	0.0523 ± 0.00793a	0.0518 ± 0.00343a	0.0458 ± 0.00423a	0.0304 ± 0.000158b	0.0559 ± 0.00215a
LsTop1	0.0306 ± 0.00163b	0.0264 ± 0.00778b	0.0333 ± 0.00527b	0.0509 ± 0.00445a	0.0214 ± 0.00426b	0.0342 ± 0.00418b
LsTFIIS	0.11 ± 0.0067b	0.0523 ± 0.00277c	0.152 ± 0.00495a	0.15 ± 0.00806a	0.097 ± 0.00845b	0.162 ± 0.0143a
Ls5.8S	529 ± 33.6e	1430 ± 61.4b	837 ± 146d	2360 ± 53.8a	493 ± 0.477e	1130 ± 38.7c
Ls5.8S-IS	113 ± 0.384b	111 ± 2.08b	181 ± 15.7a	195 ± 9.62a	114 ± 3.77b	182 ± 1.22a
LsPCNA	0.0213 ± 0.00325c	0.0411 ± 0.0061bc	0.0847 ± 0.00737a	0.0939 ± 0.00648a	0.0515 ± 0.00154b	0.111 ± 0.0164a
